# PKC and PKN in heart disease

**DOI:** 10.1016/j.yjmcc.2019.01.029

**Published:** 2019-03

**Authors:** Valeria Marrocco, Julius Bogomolovas, Elisabeth Ehler, Cristobal G. dos Remedios, Jiayu Yu, Chen Gao, Stephan Lange

**Affiliations:** a Division of Cardiology, School of Medicine, University of California-San Diego, La Jolla, USA; b Department of Cognitive and Clinical Neuroscience, Central Institute of Mental Health, Medical Faculty Mannheim, Heidelberg University, Mannheim, Germany; c Randall Centre for Cell and Molecular Biophysics, School of Basic and Medical Biosciences, School of Cardiovascular Medicine and Sciences, British Heart Foundation Research Excellence Centre, King's College London, New Hunt's House, Guy's Campus, London SE1 1UL, UK; d Bosch Institute, Department of Anatomy, University of Sydney, Sydney. Australia; e Key Laboratory of Cell Differentiation and Apoptosis of Chinese Ministry of Education, Department of Pathophysiology, Shanghai Jiao Tong University School of Medicine, Shanghai, China; f Division of Molecular Medicine, Department of Anesthesiology, David Geffen School of Medicine at UCLA, University of California-Los Angeles, Los Angeles, USA; g University of Gothenburg, Wallenberg Laboratory, Department of Molecular and Clinical Medicine, Institute of Medicine, Gothenburg, Sweden

**Keywords:** PKC, PKN, Kinase, Cardiomyopathy, Phosphorylation, ACC, anti-parallel coiled-coil, ANF, atrial natriuretic factor, ANKRD, ankyrin repeat domain protein, DAG, diacylglycerol, DGK, diacylglycerol kinase, DCM, dilated cardiomyopathy, HCM, hypertrophic cardiomyopathy, HR1, protein kinase C-related kinase homology region 1, IPP1, protein phosphatase inhibitor 1, I/R, ischemia/reperfusion, MI, myocardial infarction, MLP, muscle lim protein (also known as Csrp3), PB1, Phox and Bem1, PHLPP, pleckstrin homology domain leucine-rich repeat protein phosphatases, PKC, protein kinase C, PKN, protein kinase N, PLN, phospholamban

## Abstract

The protein kinase C (PKC) and closely related protein kinase N (PKN) families of serine/threonine protein kinases play crucial cellular roles. Both kinases belong to the AGC subfamily of protein kinases that also include the cAMP dependent protein kinase (PKA), protein kinase B (PKB/AKT), protein kinase G (PKG) and the ribosomal protein S6 kinase (S6K). Involvement of PKC family members in heart disease has been well documented over the years, as their activity and levels are mis-regulated in several pathological heart conditions, such as ischemia, diabetic cardiomyopathy, as well as hypertrophic or dilated cardiomyopathy.

This review focuses on the regulation of PKCs and PKNs in different pathological heart conditions and on the influences that PKC/PKN activation has on several physiological processes. In addition, we discuss mechanisms by which PKCs and the closely related PKNs are activated and turned-off in hearts, how they regulate cardiac specific downstream targets and pathways, and how their inhibition by small molecules is explored as new therapeutic target to treat cardiomyopathies and heart failure.

## 1 Introduction

Kinases of the PKC and closely related PKN families play multifaceted roles during cardiac development and in the pathophysiology of many cardiovascular diseases. Their crucial function as signaling nodes during heart formation and for the etiology of heart diseases makes them appealing targets for the development of novel therapies. However, their complex regulation, diverse cardiac substrates and precise roles in the pathomechanism of the numerous cardiovascular diseases remain enigmatic.

This review aims to summarize the current understanding for the cardiac specific roles that these serine/threonine kinases play in health and cardiovascular disease. We discuss molecular features that promote their activity and signaling, effects on cardiac development and physiology, tools to evaluate their action, and aspects that promote their usage as alluring therapeutic targets.

## 2 Structure and function of the PKC/PKN families

**Fig. 1:**
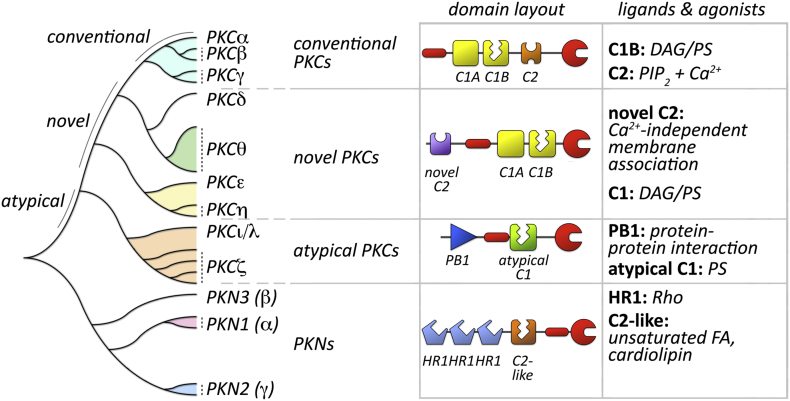
Schematic overview of the PKC and PKN families of kinases. Evolutionary analysis of human PKN and PKC kinase domains. Domain layout of conventional, novel and atypical PKCs, as well as PKN isozymes is shown, in addition to ligands, agonists or binding partners for their activation. Abbreviations: DAG – diacylglycerol; FA – fatty acid; HR1 – polybasic coiled-coil homology region 1; PB1 – Phox and Bem1; PIP_2_ – phosphatidylinositol 4,5-bisphosphate; PS – phosphatidylserine; Rho - Ras homology.

The AGC superfamily of protein kinases, which includes the cAMP dependent protein kinase (PKA), protein kinase B (PKB/Akt), protein kinase G (PKG) and the ribosomal protein S6 kinase (S6K) kinase families, as well as PDK1 (3-phosphoinositide-dependent protein kinase 1, PDPK1, also contains in one of its extended branches the protein kinase C (PKC) and the closely related protein kinase N (PKN) families (Fig. 1) [1]. Kinases in both families are marked by one or more N-terminally located regulatory domains (e.g. C1 or C2 domains in PKCs; HR1, also called ACC domains, in PKNs) that are connected by a hinge region to the C-terminally located protein kinase domain (Fig. 1). Common in both families are also the autoinhibitory elements that resemble pseudosubstrates for each of the kinases. Access to the substrate-binding cavity is either blocked when the kinase is inactive, or permitted when structural changes in the kinase remove the autoinhibitory element.

PKCs can be subdivided into three groups: the group of the conventional PKC enzymes containing PKCα, PKCβ and PKCɣ isoforms; novel PKCs consisting of PKCδ, PKCε, PKCη and PKCθ; and the group of atypical PKCs consisting of PKCζ and PKCλ/ɩ isoforms (Fig. 1).

Conventional PKCs contain two C1-domains, C1A and C1B, in their N-terminus, with C1B permitting the binding of diacylglycerol (DAG) or phosphatidylserine (PS). The C1-domain tandem is followed by one C2-domain that coordinates binding to membrane-associated phosphatidylinositol 4, 5-bisphosphate (PIP_2_) and calcium (Ca^2+^). Novel and atypical PKCs contain in their N-termini a ‘novel’ C2 domain that is Ca^2+^-independent or a Phox and Bem1 (PB1)-domain that mediates protein-protein interactions, respectively. Both PKC families retain the ability to bind to phosphatidylserine through the centrally located tandem C1-domain repeat (novel PKCs) or one atypical C1-domain (atypical PKCs). However, only novel PKCs retained the ability to be activated by DAG.

The predominant PKC expressed in cardiomyocytes is PKCα [2,3]. However, other PKC isozymes, like PKCβ, PKCδ, PKCε were also shown to be expressed at lower levels in healthy hearts, or induced during pathological cardiac remodeling [4].

PKN was originally discovered by hybridizing a cDNA fragment that encodes for the kinase domain of PKCβ2 under low stringency conditions with a hippocampal cDNA library, attesting to the high degree of homology between PKC and PKN kinases [5]. Three PKN genes give rise to PKN1 (also known as PKN, PRK1, PAK1 or PKNα), PKN2 (PRK2, PKNβ) and PKN3 and their respective splice isoforms. The PKN catalytic domains share approximately 50% sequence identity with the PKC family members. However, the N-terminal regulatory regions are radically different between the PKN and PKC kinases. All PKN family members exhibit three unique polybasic coiled-coil homology region 1 (HR1)-domains (also called antiparallel coiled-coil (ACC)-domains), which confer binding and regulation by the Rho family of small GTPases as well as a lipid regulated autoinhibitory C2-like domain [[6], [7], [8]]. PKN1 and PKN2 are the two major isoforms of PKN in cardiomyocytes [9,10].

### 2.1 Activation of the kinase

**Fig. 2:**
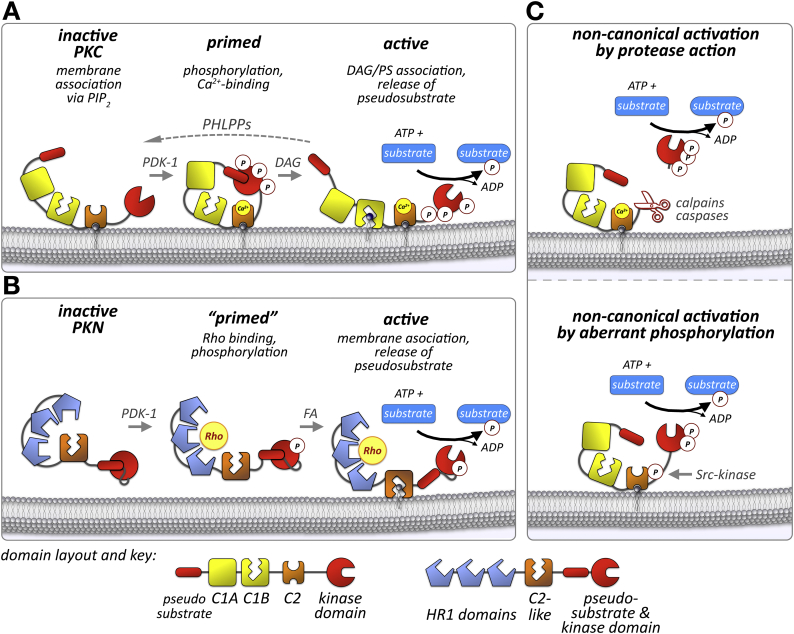
Activation of PKCs and PKNs. A-B. Schematic steps for the canonical activation of prototypical conventional PKC isozymes (A) or PKNs (B) are illustrated. C. Non-canonical activation by protease activity or tyrosine phosphorylation.

The activation of PKCs and PKNs is a very complex process, involving membrane-association of the enzymes, priming by phosphorylation, conformational changes induced by binding of proteins or second messengers (e.g. Ca^2+^, phosphatidylserine), and the release of a pseudosubstrate to allow substrates access to the catalytic cleft within the kinase domain (Fig. 2).

#### 2.1.1 Pseudosubstrates

Both PKC and PKN families contain a stretch of basic amino acids that act as pseudosubstrates and block access to the catalytic cleft of the kinase when the enzyme is inactive. Amino acids contained in the pseudosubstrate sequence resemble the consensus substrate sequence for the kinase, but with an alanine in place for the serine/threonine residues. The location of the pseudosubstrate in PKCs is upstream to the C1-domain(s) [11], whereas in PKNs it is found in the hinge-region N-terminal to the kinase domain itself [8]. Pseudosubstrate sequences are able to effectively inhibit kinase activity in the full-length context, and are typically released from the catalytic cleft of the kinase domain in a conformation dependent manner that relies on posttranslational modifications and/or ligand binding. Isolated pseudosubstrate peptide sequences were shown to have comparatively low affinities, e.g. the IC_50_ of the PKCα pseudosubstrate (amino acids 19–27) is ~6 μM. Further experiments demonstrated that affinities could be enhanced by increasing the peptide length (e.g. increasing PKCα pseudosubstrate peptide to encompass amino acids 19–31 decreased the IC_50_ by more than tenfold to approx. 0.1 μM), and mutational analysis demonstrated roles for key residues (e.g. mutation of Arg27 to alanine increased the IC_50_ of the PKCα pseudosubstrate peptide [amino acids 19–31] to ~3.1 μM) [12]. However, the effectiveness of isolated pseudosubstrate peptides may be ultimately too poor to be effectively used in therapeutic approaches due to their weak affinity and reduced isoform specificity. Specifically the latter may also have some ramifications on study outcomes that utilized pseudosubstrate peptides in experiments.

#### 2.1.2 Phosphorylation

PKCs contain a series of conserved phosphorylation sites that are located within the kinase activation loop, turn-motif and hydrophobic motif. While phosphorylation at these sites is constitutive to mature and prime the kinase as well as enhance its cellular half-life, they are generally not thought to represent the activity of the kinase. Initial phosphorylation of PKCs and PKNs in the activation loop is done by the 3-phosphoinositide-dependent kinase 1 (PDK1). For PKCs, this phosphorylation entails the help of molecular chaperones Hsp90 and Cdc34, as well as TORC2 (target of rapamycin complex 2) [[13], [14], [15]], while it was reported that PDK1 dependent phosphorylation of PKN1 is enhanced by prior binding of activated RhoA [16]. Following this step, PKCs are subsequently phosphorylated in the turn and hydrophobic motif, either by auto-phosphorylation [17], or actions of other kinases, such as mTOR (mammalian target of rapamycin) [18], Src-family kinases, phosphatidylinositol 3-kinases (PI3K) [19], or other PKC isozymes [20]. While less is known for PKNs, it has been demonstrated that PKN2 may be activated by MEK kinase 2 (MEKK2), a mitogen-activated protein kinase [21]. More recent studies using mass-spectrometry based phospho-proteome profiling identified a plethora of other phosphorylation sites within PKCs and PKNs, including conserved phospho-tyrosine sites [22,23]. It remains to be determined if these sites influence the activity or kinase half-life, and which upstream kinases are responsible for their phosphorylation.

#### 2.1.3 Ligands and protein binding partners

Prototypical conventional PKCs are regulated by binding of the C1B-domain to either DAG or phosphatidylserine, as well as association of PIP_2_ and the second messenger Ca^2+^ with the C2-domain. The affinity of these domains is tuned by intramolecular conformational changes that initially mask the C1- and C2-domains from the association with their agonists. Unmasking is induced by phosphorylation events, and calcium-induced membrane association. Some of the regulatory steps that require calcium in conventional PKCs have been replaced with the prerequisite for calcium-independent membrane associations [24,25] or obligatory protein-interactions in novel and atypical PKCs, which however retained the necessity to associate with DAG and/or phosphatidylserine. The dependence on lipids or membrane components is also conserved for PKNs, which can be activated by unsaturated fatty acids such as arachidonic acid, linoleic acid, cardiolipin [25], phosphatidylinositol phospholipids (PI(4,5)P_2_, PI(3,4,5)P_3_), lysophosphatidic acid or lysophosphatidylinositol [26].

In addition to the lipid or membrane component, atypical PKCs and PKNs require dedicated protein interaction partners to become fully active. Examples include interaction of p62 or Par6 with the PB1 domain in atypical PKCs [[27], [28], [29]]. PKNs require binding of small GTPase proteins of the Ras homology gene family (RhoA, RhoB or RhoC), which highlights their involvement in cytoskeletal reorganization [[6], [7], [8]]. PKN2 was also demonstrated to be activated by the small GTPase Rac [30,31].

#### 2.1.4 Non-canonical activation of PKCs and PKNs

More recently, several pathways emerged to activate PKC or PKN that do not rely on canonical co-factors or ligands. It has been known for some time that active proteases, such as calpain or caspase can cleave the catalytic domain off the regulatory elements, thereby allowing the kinases to become active [[31], [32], [33], [34], [35]]. However, the catalytic activity of these proteases is tightly controlled in healthy cells, suggesting that this mechanism of kinase activation may be more relevant for cells undergoing apoptosis, or during pathological remodeling [[36], [37], [38]]. Once activated however, these ‘rogue’ kinases are most often very detrimental for cardiac health, as transgenic mice expressing a PKCα cleavage fragment illustrate [39]. Another non-canonical pathway to overcome inhibition of kinase activity by its regulatory elements was shown during oxidative stress, where PKCδ was activated by Src-mediated phosphorylation of an otherwise not utilized tyrosine residue in the enzyme [40,41].

#### 2.1.5 Receptors for activated C-kinase (RACKs) and other proteins and posttranslational modifications contributing to kinase activation and function

In addition to the well-characterized ligands, second messengers and binding-partners (e.g. p62, Par6, Rho family members) that are required for activation of kinases, PKCs and PKNs are also modulated by signaling pathways and interactions with other proteins.

##### 2.1.5.1 MLP and muscle ankyrin repeat proteins

Intriguingly, it recently emerged that muscle lim protein (MLP/Csrp3) and muscle ankyrin repeat proteins Ankrd1 (CARP1) and Ankrd2 (CARP2) may play more direct roles in the regulation of PKC activity. Using *in vitro* kinase assays it was shown that MLP and proteins from the muscle ankyrin repeat protein family are substrates of PKCα [42]. Specifically for MLP it was striking to note that a) increased phosphorylation of MLP correlates with dilated cardiomyopathy (DCM) in patients, and b) MLP mutations associated with hypertrophic cardiomyopathy (HCM) result in decreased phosphorylation, while mutations associated with DCM development displayed markedly increased MLP phosphorylation [42]. In addition, *in vitro* kinase assays indicated that MLP may act as a direct inhibitor of PKCα activity through a negative feedback loop mechanism [42]. Increased PKCα levels and activity in the hearts of MLP knockout mice underscore this finding. Moreover, MLP knockouts also revealed that muscle ankyrin repeat proteins Ankrd1 and Ankrd2 are directly involved in the pathological activation of PKCα, sequestering PKCα with phospholipase C at intercalated disks. Indeed, deletion of Ankrd1 or Ankrd2 in MLP knockout mice prevented DCM development [42].

##### 2.1.5.2 RACKs

While Ankrd1 and Ankrd2 are two examples that influence the temporal and spatial regulation of kinase activity, other proteins that ‘scaffold’ or ‘anchor’ kinases to certain cellular compartments or macromolecular complexes have been known for a long time. One such family are the RACK (receptors for activated C-kinase) proteins, which are thought to be partially responsible for the various subcellular localizations of the activated isozymes within cardiomyocytes [43]. Rack1 (also known as guanine nucleotide-binding protein subunit β-2-like 1, Gnb2L1) was shown to have preference for binding to PKCβ, PKCδ and PKCε, as compared to PKCα or PKCɣ [44,45], while Rack2 (better known as coatomer subunit β, CopB2) is thought to primarily associate with PKCε [46]. Both Rack proteins were shown to bind to active PKCs, and its was demonstrated that Rack2 directs the subcellular localization of PKCε to the Golgi apparatus in cardiomyocytes [44,46]. Intriguingly, Rack2 was shown to associate with myofilaments and intercalated discs in cultured neonatal cardiomyocytes, while Rack1 displayed perinuclear staining [46,47]. The importance of Rack interactions for PKC function was demonstrated in a study that investigated transgenic mice expressing PKCε, showing distinct cardiac phenotypes that depend on the expression levels of Rack proteins [48].

##### 2.1.5.3 Other kinase-binding proteins and modifications known to modulate kinase activity

Another protein that associates with PKCs and is thought to regulate their activity is PICOT (Protein kinase C-interacting cousin of thioredoxin; GLRX3), which was shown to bind via its N-terminal thioredoxin homology domain to the kinase domains of PKCζ and PKCθ [49,50]. Several studies link PICOT function to the modulation of cardiac hypertrophy and contractility [[50], [51], [52]]. Crucial cardiac functions of PICOT for inhibiting PKCθ activity are underscored when looking at PICOT global knockout mice, which display hemorrhages in the head and result in embryonic lethality between embryonic days E12.5 and E14.5 [51]. However, it is unclear if this finding can be reproduced in cardiac specific knockouts for PICOT. Further studies using PICOT transgenic mice and heterozygous knockouts revealed important functions for PICOT in ischemic/reperfusion (I/R), with decreased PICOT levels resulting in attenuated I/R injury and reactive oxygen-species production [53].

While the biological functions of PKC phosphorylation have long been characterized, it recently emerged that at least one PKC isozyme, PKCζ, may also undergo posttranslational modification by lysine-acetylation [54]. The authors of this study found that the deacetylase SIRT1 represses PKCζ activation by inhibiting its initial PDK1 mediated phosphorylation. This mechanism of kinase regulation may be important for the pathogenesis of cardiac hypertrophy, although exact molecular mechanisms, i.e. which lysine residues in PKCζ undergo modification by acetylation, remain to be discovered.

### 2.2 Role of PKCs and PKNs in normal cardiac development and in disease

#### 2.2.1 Conventional PKCs

**Fig. 3:**
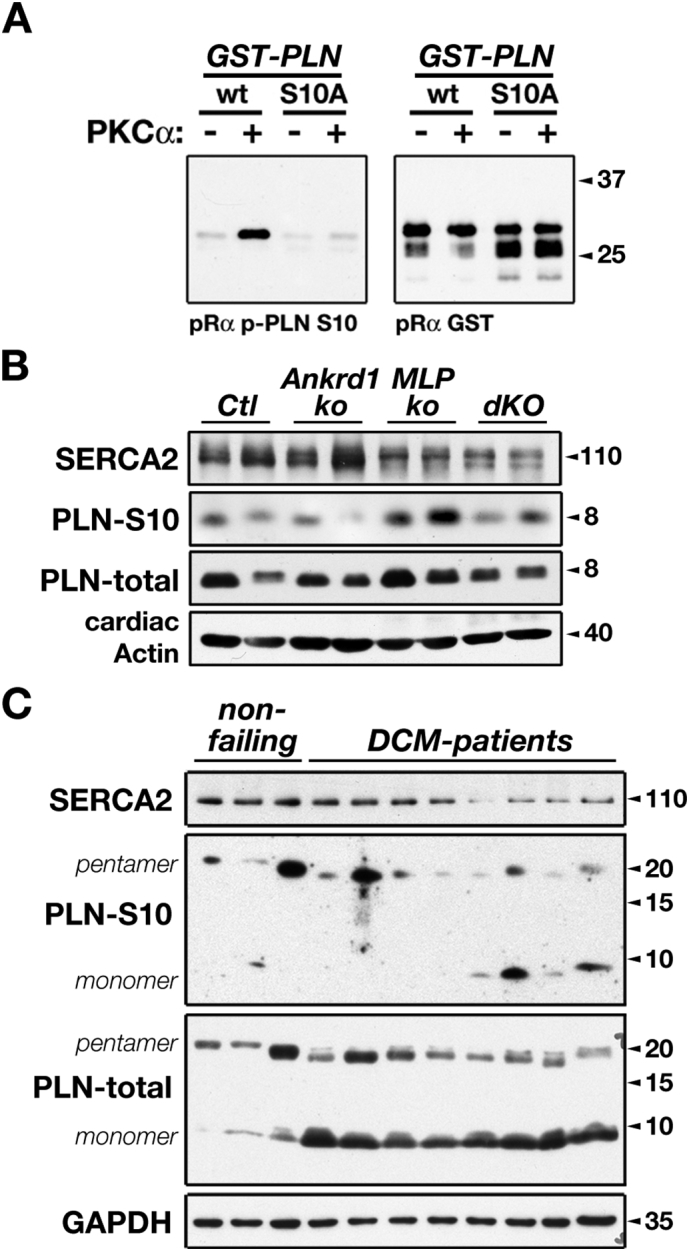
Phospholamban phosphorylation at Ser10. A. Verification of the specificity of the phospholamban (PLN) Ser10 antibody using *in vitro* kinase assay-mediated phosphorylation of wildtype (wt) or Ser10Ala mutant (S10A) GST-PLN fusion proteins. Only phosphorylation at Ser10, but not S10A mutant PLN is recognized by the antibody. B. Analysis of SERCA2, total and phospho-Ser10 PLN levels in total cardiac extracts of control (Ctl), Ankrd1 knockout (Ankrd1-ko), MLP knockout (MLP-ko) or Ankrd1/MLP double knockout (dKO) mice indicates increased monomeric PLN phosphorylation at Ser10 in MLP-ko. Cardiac actin was used as loading control. C. Analysis of SERCA2, total and phospho-Ser10 PLN levels in cardiac extracts from non-failing individuals and dilated cardiomyopathy patients. Molecular weights of monomeric and pentameric phospholamban species are indicated, and reveal increased monomeric phosphorylation of PLN at Ser10 in patients with pathologically decreased SERCA2 levels. GAPDH was used as loading control.

Among the different PKC isozymes expressed in cardiac tissue, PKCα is the predominant member [2,3,55]. This central role for PKCα is underscored by many studies that investigated PKCα functions in the heart. As a consensus it emerged that PKCα is increased in human cardiac pathology [55,56] and during the transition to heart failure [57], plays crucial roles for cardiomyocyte hypertrophy, and as an important regulator of muscle contractility [58,59]. While knockout mouse for PKCα show increased cardiac functional performance and a normal hypertrophic response to pressure overload, cardiac specific overexpression resulted in cardiac hypertrophy around 6–8 months of age with a reduced fractional shortening at 4 months [58]. PKCα is able to phosphorylate a plethora of cellular substrates, and several of its cardiac specific substrate proteins were identified. Among the best-characterized substrates with cardiac roles are protein phosphatase inhibitor-1 (IPP1), the α1C-subunit of the L-type calcium channel, troponin complex proteins or titin (Table 1). Specifically IPP1 phosphorylation by PKC has been characterized as modulator for protein phosphatase-1 activity, indirectly affecting phospholamban (PLN) phosphorylation and its modulatory role for sarco(endo)plasmic reticulum Ca^2+^-ATPase (SERCA) action. While PLN itself has also been characterized as a PKCα substrate *in vitro* [60,61], analysis of PLN after PKCα stimulation by PMA in intact healthy myocardium did not reveal enhanced phosphorylation levels [62]. This result discounted direct roles for PKCα function on PLN *in vivo*. However, the availability of a Ser10 specific PLN antibody (Fig. 3A) revealed possible roles for this site in dilated cardiomyopathy and heart failure. Our analysis of PLN phosphorylation in muscle lim protein (MLP) knockout mice, a model for dilated cardiomyopathy with known pathological involvement of PKCα, does show increased phosphorylation levels of monomeric PLN at Ser10 (Fig. 3B; monomeric PLN, as opposed to its pentameric version is thought to modulate SERCA activity). In addition, PLN phosphorylation levels in MLP/Ankrd1 double knockout mice that show normalized PKCα signaling [42] display reduced PLN phosphorylation levels at this site. Analysis of human cardiac biopsies from healthy donors and DCM patients also revealed disease-related changes to monomeric PLN phosphorylation at Ser10, specifically in patients that show pathologically decreased SERCA expression levels (Fig. 3C). This result warrants further analysis of PLN phosphorylation at this site in cardiac pathology. In addition, investigations into functional effects on SERCA activity mediated by this posttranslational modification would shed further mechanistic insights. This is underscored by the fact that residues surrounding Ser10 were shown to be important for the recognition of PLN by PKA [63,64].

**Table 1:** Select PKC substrates and characterized phosphorylation sites with cardiac roles.

Kinase family	Cellular category	Select substrates and phosphorylation sites (if known)	Cardiac roles	References
PKCs	myofibrillar	**myosin light chain-2** (MLC2v; rat Ser15)	modulation of force generation	[166]
		**titin** (PEVK region; Ser11878, Ser12022)	modulation of titin compliance	[167]
		**muscle lim protein** (MLP, CSRP3)	modulation of PKC activity	[42]
		**muscle ankyrin repeat proteins** (Ankrd1/CARP1, Thr11, Thr116, Ser305; Ankrd2/CARP2; Ankrd23/CARP3)	unknown, potential regulation of stretch-responsive signaling pathways	[168]
		**troponin-I** (Ser42, Ser44, Ser76, Ser198, Thr143, Ser198)	modulation of force generation	[[169], [170], [171], [172], [173], [174], [175]]
		**troponin-T** (Ser1, Thr194, Ser198, Thr203 and Thr284, Ser179)		
		**myosin binding protein-C** (MyBPC, Ser275, Ser302, Ser304)	modulation of force generation	[69,166]
	ion channels & pumps, membrane-associated proteins	**L-type calcium channel Ca(v)1.2** (α1C-subunit, Ser1674, Ser1928)	increased channel activity	[59,176]
		**Connexin-43** (Cx43, Ser368)	regulation of subcellular Cx43 distribution	[177]
		**K(v)1.5 channel** (Kvβ1.3 regulatory subunit)	modulation of channel activity	[70]
		**ryanodine receptor** (RyR)	modulation of channel activity	[178]
		**sarcoendoplasmic reticulum Ca**^**2+**^**-ATPase** (SERCA)	modulation of SERCA activity	[179]
		**phospholamban** (PLN, Ser16, Ser10?)	modulation of SERCA activity, unknown roles for Ser10 phosphorylation	[60,61]
		**vinculin** (Ser1033, Ser1045)	modulation of lipid-docking	[180]
	signaling	**β2-adrenergic receptor** (β2-AR; Ser261, Ser262, Ser344, Ser345)	modulation of adrenergic signaling & force generation	[181]
		**G-protein coupled receptor kinase** (GRK2, Ser29; GRK5)	enhances GRK activity, modulation of adrenergic signaling	[182,183]
		**histone deacetylase 5** (HDAC5; Ser259, Ser498)	modulation of nuclear shuttling and transcriptional activity	[34,184]
		**signal transducer and activator of transcription 3** (STAT3, Ser727?)	modulation of gene expression	[88]
		**protein phosphatase inhibitor-1** (IPP1; Ser67)	modulates protein phosphatase-1 activity, indirectly influences PLN and SERCA activity	[58]
	metabolism & mitochondria	**glyceraldehyde 3-phosphate dehydrogenase** (GAPDH, Thr246)	modulation of GAPDH-driven mitophagy in ischemia/reperfusion	[185]
		**subunit IV of the cytochrome c oxidase complex** (COIV)	modulation of COIV activity	[186,187]
		**Dynamin-Related Protein 1** (Drp1**;** Ser616)	modulation of mitochondrial fission	[188]
		**pyruvate dehydrogenase kinase 2** (PDK2)	modulation of enzyme activity	[189]
		**6-phosphofructokinase-2** (PFK2, Thr475)	modulation of enzyme activity	[90]
PKNs	myofibrillar, cytoskeletal	**α-actinin 2** (ACTN2)	unknown, potential regulation of actin cytoskeleton	[104]
		**β-adducin** (ADD2)	unknown, potential role in hypertension and dilated cardiomyopathy	[105,190,191]
		**α-B crystallin** (CryAB)	unkown, potential role during ischemia	[95]
	signaling	**Focal adhesion kinase 1** (FADK1/PTK2)	unknown, potential role for integrin signaling	[105]
		**G-protein coupled receptor kinase** (GRK4)	unknown, potential modulation of G-protein coupled receptor signaling	[105]
		**oxidative stress responsive kinase 1** (OXSR1/OSR1)	unknown, potential role during cardiac development	[105,192]
		**serine/arginine-rich specific kinase 3** (SRPK3/STK23)	unknown, potential role in ischemia and dilated cardiomyopathy	[105,193,194]
		**mammalian sterile 20-like kinase 1** (MST1/STK4)	unknown, potential role in ischemia and autophagy regulation	[105,195,196]
		**mammalian sterile 20-like kinase 2** (MST20/STK3)	unknown, potential modulation of cardiac hypertrophy	[105,197]
		**protein kinase C ζ** (PKCζ)	unknown	[105]
	other	**Family with sequence similarity 64, member A** (Fam64A)	unknown, potential regulation of cardiomyocyte proliferation in hypoxia	[105,198]

Analysis of cardiac functions for PKCβ and PKCɣ revealed that they appear to play minor roles, as deletion of both genes in mice showed negligible cardiac phenotypes [65]. During cardiac pathology however, PKCβ is upregulated, a result that suggests roles for this PKC isozyme in cardiomyopathy [55,56]. Loss of all conventional isozymes in mice had no attenuating effect on pressure overload-induced hypertrophy. However, long-term transverse aortic constriction showed that PKCβ/ɣ double knockouts developed a more severe form of heart failure. These and other reports using transgenic mice and other mouse models [[66], [67], [68]] indicate that increased expression of at least PKCβ may be beneficial rather than detrimental for cardiac physiology.

#### 2.2.2 Novel PKCs

Studies investigating novel PKCs in the heart have largely focused on their role during ischemia-reperfusion injury, centering on molecular functions in mitochondria. However, novel PKC isozymes have also been shown to be able to phosphorylate substrates outside mitochondria, including myosin-binding protein-C (MyBPC) or the regulatory subunit of the voltage gated potassium channel (Kv1.5) [69,70].

Knockout mice for PKCθ develop dilated cardiomyopathy accompanied by reduced fractional shortening, increased fibrosis, and enhanced levels of cardiomyopathy markers such as ANF, as well as activation of p38 and JNK pathways [71]. In contrast to the dramatic phenotype of PKCθ knockouts, genetic ablation of PKCδ and/or PKCε did not result in a profound postnatal cardiac baseline phenotype [[72], [73], [74], [75]]. However, PKCδ knockouts display dramatic changes to the cardiac metabolism, as glycolytic enzymes were dramatically reduced, while enzymes involved in β-oxidation and lipid metabolism were significantly enhanced. Loss of both isozymes during postnatal development resulted in transcriptional changes typical of pathological hypertrophy [75]. In addition, lack of both isozymes during embryonic cardiac development resulted in lethality in the majority of double-knockout embryos, which was marked by abnormal hypertrophy and severely compromised or abolished ventricular cavities.

Looking at their role in myocardial infarction, PKCδ and PKCε appear to play opposing roles during ischemia and reperfusion. While PKCδ activity is thought to promote reperfusion injury following ischemia [76], PKCε was found to be protective, when activated during preconditioning [77]. However, data on the role of PKCδ in the heart are controversial. While some studies suggest PKCδ inhibition during reperfusion may alleviate injury-associated pathology, PKCδ knockouts did not protect but exaggerated the ischemia-reperfusion injury after preconditioning [78]. In addition, preconditioned PKCδ knockouts presented with a bigger infarct size compared to groups not treated with preconditioning.

The cardiac role of PKCη remains to be explored. Despite being described as specifically expressed in heart [79], the heart phenotype of PKCη knockout mice has not been described [80,81].

#### 2.2.3 Atypical PKCs

Little is known about cardiac roles for atypical PKCs. PKCɩ (the human homolog of PKCλ in mice) was shown to play important roles for embryonic heart formation. Cardiac specific embryonic knockout mice show no visible trabeculations, caused by depolarized cellular divisions of luminal myocardial cells [82]. Postnatal muscle specific PKCλ knockout mice (driven by the MCK-Cre promoter [83]) show impaired glucose uptake and other features of metabolic syndrome [84,85]. However, these studies also revealed that PKCλ and PKCζ may have interchangeable functions for glucose and fatty acid uptake [85]. In addition, infusion of PKCɩ restored Ca^2+^ entry through the voltage-dependent L-type Ca channel (CaV1.2) in a diabetic mouse model [86], indicating this cardiac channel as a potential substrate for this kinase.

Despite reports that PKCɩ/λ and PKCζ have interchangeable functions for the metabolic state of cardiomyocytes, PKCζ was shown to perform unique and diverse functions in the heart. Knockouts for PKCζ appear normal with no reported cardiac abnormalities in baseline [87]. However, it was shown that activation of PKCζ in cardiomyocytes promoted their hypertrophy via activation of signal transducer and activator of transcription 3 (STAT3) [88], and loss of PKCζ in mice resulted in impaired hypertrophic signaling through Gq-dependent ERK5 activation following angiotensin-II treatment [89]. In addition, *in vitro* studies demonstrated that PKCζ is able to phosphorylate 6-phosphofructose-2 kinase (PFK2), indicating that this PKC isozyme plays a role for the glycolysis in the heart [90].

#### 2.2.4 PKNs

Published results have demonstrated that PKN1 and/or PKN2 protein levels and/or activity are deregulated in different cohorts of human cardiac pathologies such as atrial fibrosis [91], hibernating myocardium [92] or dilated cardiomyopathy [93], act as cardiomyocyte survival factors after I/R injury [[94], [95], [96]] and can regulate ANF expression in vitro [96]. Moreover, patients suffering from 19p13.12 microdeletion syndrome, which results in the loss of the PKN1 gene, are characterized by congenital heart abnormalities and arrhythmias [97,98]. However, most of our knowledge about the function of PKNs in the heart has been from in vitro studies utilizing cultured cells and their roles in cardiac pathophysiology *in vivo* are not well understood. Because PKN1 and PKN2 are expressed beyond the heart [99], the published mouse models have not adequately addressed the cardiomyocyte-specific function of PKN1/2. Loss of PKN1 in 19p13.12 microdeletion syndrome supports the importance of PKN1 in cardiovascular development. Global knockouts for PKN1 and the more homologous PKN3, as well as PKN1/3 double knockouts displayed no overt cardiovascular phenotype [94,100]. However, PKN1 knockouts did exhibit larger infarcts in response to MI, suggesting that endogenous PKN1 provides cardioprotection *in vivo* [94]. In addition, evidence indicates that PKN1 is not only involved in cardioprotection upon I/R, but it is also involved in mediating pro-hypertrophic signaling. Global loss of PKN2 in mice results in growth, morphogenetic, and cardiovascular defects and is embryonically lethal by day E10.5. Characterization of global PKN2 null mice concluded that PKN2 is required for developmental mesoderm growth and neural crest migration *in vivo*. Interestingly, conditional PKN2 deletion driven by sm22α-Cre that is partially active in cardiomyocytes [101] was not lethal *in utero*. These animals were born but presented prominent cardiovascular pathology [100], which intriguingly contrasts with global PKN2 knockout mice. Previous studies have shown that A Kinase Anchoring Protein (AKAP-Lbc) assembles a signaling complex composed of the kinases PKN1, MLTK, MKK3 and p38α that mediates the activation of p38 in cardiomyocytes in response to aortic banding-induced pressure overload or adrenergic stimulation [102,103]. PKNs can also be activated by lipids via the C2-like domain, Rho GTPases via the Hr1 domain and even by caspase cleavage, although the *in vivo* significance is as yet not fully characterized [[6], [7], [8],^31^]. However, it is not understood how these various molecular pathways are integrated in cardiac PKN signaling.

Furthermore, the molecular mechanisms by which PKN1 mediates cardioprotection remains to be addressed. Whether PKN1/PKN3 and PKN2 have distinct and overlapping roles in cardiac muscle is also not understood.

There have been less than 20 protein substrates identified for PKNs (Table 1), and for the majority of them physiological relevance of their phosphorylation by PKNs has not been yet confirmed [104,105]. However, it was shown that PKN can be involved in the regulation of important cardiac molecules and structures including atrial natriuretic factor (ANF) [106], αB-crystallin [107], calcium/calmodulin-dependent protein kinase (CamKIIδ) and phospholamban (PLN) [94]. Moreover, it is striking to note that among the PKN substrates are several protein kinases with known cardiac roles, including mammalian sterile 20-like kinase 1 and 2 (MST1, MST2) or PKCδ, as well as sarcomeric α-actinin [104,105]. Specifically, the phosphorylation of several kinase classes and families indicates another problem faced when analyzing cardiac roles for PKCs and PKNs: their functional redundancy and their high degree of ‘cross-talk’, which needs to be evaluated in each condition, animal model and disease.

### 2.3 Inactivation of the kinase

Kinase activity is tightly controlled within healthy cells. While it is important to discuss agonists, ligands and cofactors that contribute to the activation of PKCs and PKNs, it is also good to keep in mind that deactivation of kinases is equally controlled to maintain proper cellular homeostasis. A first step to deactivate PKCs is the active removal of agonists like calcium or DAG by sequestration or chemical conversion. Diacylglycerol kinases (DGKs) phosphorylate DAG to generate phosphatidic acid, thereby removing it from the bioavailable pool and lowering PKC activity. The 10 mammalian DGKs can be subdivided into five classes, each presenting with different domain structures, ways to associate with membranes, and various requirements for calcium and lipid agonists [108,109]. At least four DGKs have been reported to be expressed in heart: DGKα, DGKη, DGKϵ and DGKζ [109,110]. However, further work is required to investigate their roles during cardiac development and in disease. Diacylglycerol acyltransferase 1 (DGAT1) is another enzyme that lowers bioavailable DAG to triglyceride, and has recently been shown to play important functions for the heart. Loss of DGAT1 in hearts results in chronically increased DAG and ceramide levels, leading to heart failure and death in 50% of mice by 9 months of age [111].

Dephosphorylation of PKCs is mediated by several protein phosphatases, most notably by the family of pleckstrin homology domain leucine-rich repeat protein phosphatases (PHLPPs), protein phosphatase 1 (PP1A), protein phosphatase 2A (PP2A) or protein phosphatase 2B (PP2B/calcineurin) [[112], [113], [114], [115], [116]]. However, increased phosphatase levels do not necessarily translate to enhanced dephosphorylation of PKCs, as seen in PP2B/calcineurin transgenic mice that paradoxically display increased PKC activity [117]. Although little is known about specific phosphatases that contribute to the dephosphorylation of PKNs, PP2A was shown to serve this function [118]. However, the action of these phosphatases is not exclusively directed towards PKC or PKN: e.g. PHLPPs have been demonstrated to also mediate the dephosphorylation of other members of the AGC family of kinases, such as AKT/PKB or p70S6K [113,119]. In addition, the dephosphorylation of protein kinases may be aided by isomerases, such as the Peptidyl-prolyl cis-trans isomerase NIMA-interacting 1 (Pin1), which was demonstrated to be required for efficient PKC dephosphorylation [120].

Degradation of PKCs involves actions of the ubiquitin proteasome system and non-proteasomal pathways [121]. This result indicates the presence of multiple pathways to regulate cellular kinase levels. The muscle specific E3-ubiquitin ligase RINCK/Trim41 (RING-finger protein that interacts with C kinase) was identified in yeast-two hybrid assays as interaction partner for the C1A domain in PKCβ [122]. Further experiments indicated that RINCK is able to poly-ubiquitylate isozymes from all PKC families, albeit with weaker affinity for atypical PKCζ compared to conventional or novel PKCs. However, RINCK was shown to promote the ubiquitylation of PKC independently of the phosphorylation state of the kinase. Another study found preference for the degradation of phosphorylated PKCs by a proteasome-dependent pathway, again underscoring the presence of multiple degradation mechanisms [121]. Other E3-ubiquitin ligases that have been reported to interact with PKC isozymes and promote their ubiquitylation are HOIL-1 L (heme-oxidized IRP2 ubiquitin ligase 1 L) and HOIP [121,123], or CHIP (carboxyl terminus of constitutive heat shock cognate 70 (HSC70)-interacting protein) [124]. However, their exact role in the regulation of PKCα and other PKCs in the heart remains to be determined.

A recent study investigated the sequential posttranslational modifications of PKCα before degradation of the kinase can occur [125]. Specifically, the study highlights the emerging role that SUMOylation plays for the prevention of premature degradation of PKCα. The authors argue that by reducing the availability of key-lysine residues for modification by ubiquitin, PKCα remains longer active and less prone to be targeted for degradation. However, the effect of PKC SUMOylation is controversial, as another report indicated that enhanced SUMOylation of PKC inhibited its activity [126]. Further analysis and identification of involved E3-ligases responsible for kinase SUMOylation, such as Nup358 [127] or PIASxβ [128] is needed to evaluate the role that this posttranslational modification plays for kinase activity and longevity.

### 2.4 Measurement and evaluation of PKC/PKN activity

It is challenging to accurately assess kinase activity in cells and tissues. Part of the problem are functional redundancies and signaling cross-talk between closely related members of the PKC and PKN protein kinase families, the promiscuity of serine/threonine residues in substrate proteins for a number of kinases, and the complexity of the cellular context, involving subcellular localization, binding partners and posttranslational modifications that modulate kinase activity. Nonetheless, over the years, a number of tools have been developed that helped in probing kinase activity and their biological effects (summarized in Table 2), including mouse models, (phospho-specific) antibodies, small molecule and peptide inhibitors and agonists, model substrates, subcellular translocation assays, fluorescent reporter constructs, and large-scale phospho-proteome surveys [129,130]. While these tools have been proven useful, it later emerged that some of the assumptions used to evaluate kinase activity/inhibition evolved based on novel findings. One example is the usage of peptide regulators [131] that forego the finding of non-canonical mechanisms to activate protein kinases (reviewed and commented in [4]) and were found to exhibit only modest specificity, with subtle differences between the selectivity in the targeted kinases (reviewed and commented in [132]). Similarly, the usage of small molecule kinase inhibitors needs to be critically assessed, as some PKC inhibitors like Rottlerin (Mallotoxin) were found to exhibit significant off-target effects [133]. Indeed, large-scale kinase inhibitor screens revealed how complex and manyfold off-target effects of small molecule inhibitors can be, even for molecules that are widely considered to be very selective [134,135].

**Table 2:** Select mouse models, kinase activity reporters and inhibitors.

Category	Kinase, effectors & inhibitors	Cardiac Phenotype/Comments	References
Mouse models	PKCα	Knockout:	[58]
		- PKCα knockout avert progression from hypertrophy to heart failure	
		Transgenic mice:	[58]
		- overexpressing mice have hypercontractile hearts - peptide inhibitor/activator	
	PKCβ/ɣ	Knockouts:	[65,67,199]
		- PKCβ and PKCɣ single knockouts display no overt baseline cardiac phenotype - PKCβ knockout display decreased infarct size and enhanced recovery of left ventricular (LV) function - deletion of both genes in mice resulted in negligible cardiac phenotypes	
		Transgenic mice:	[66,68,200]
		- PKCβ overexpression causes hypertrophy & sudden death - low level PKCβ overexpression improved recovery from I/R - conditionally overexpressing PKCβ mice display increased cardiac contractility and altered calcium transients	
	PKCδ/ε	Knockouts:	[[72], [73], [74], [75],^78^]
		- Loss of PKCδ results in metabolic changes - Knockout mice for PKCε show higher susceptibility for I/R injury - Postnatal specific PKCδ/PKCε double knockouts had normal-sized hearts, however displayed increase in hypertrophy markers and exacerbated hypertrophy and cardiac dysfunction when challenged - Loss of both isozymes in utero resulted in embryonic lethality, marked by abnormal hypertrophy and reduced ventricular cavities	
		Transgenic mice:	[48,201,202]
		- PKCδ gate-keeper mutant knock-in mice (AS-PKCδ) - overexpression causes concentric cardiac hypertrophy - overexpression of A/E constitutively active PKCθ display protection from I/R-injury or cardiac hypertrophy and heart failure (depending on the expression level)	
	PKCθ/PKCeta	Knockouts:	[71,80,81,203]
		- PKCθ knockouts develop dilated cardiomyopathy, highlighting the role of the kinase for cardiomyocyte survival and remodeling - cardiac phenotype of PKCη knockouts has not been characterized	
		Transgenic mice:	[204]
		- muscle specific K/R (kinase dead) overexpressing mice (uncharacterized cardiac phenotype)	
	PKCλ/ɩ	Knockout:	[82,84,85]
		- global knockouts are embryonically lethal - cardiac specific embryonic knockouts display trabeculation-defects - muscle specific postnatal knockouts display signs of metabolic syndrome	
	PKCζ	Knockout:	[87,89]
		- PKCζ knockouts display no baseline cardiac phenotype, but impaired induction of cardiac hypertrophy by angiotensin II	
	PKN1/PKN3	Knockouts:	[94,100]
		- no overt cardiac phenotype in PKN1/PKN3 single and double knockouts - global PKN1 knockouts develop mild systolic and diastolic dysfunction	
	PKN2	Knockout:	[100]
		- Global knockouts are lethal at E10, displaying cardiovascular and morphological defects - tissue-specific PKN2 knockout (SM22α Cre) show partial lethality, escapers develop cardiac hypertrophy	
Evaluation of kinase activity	direct measurement of PKC/PKN activity *in vitro*	PKC translocation from the cytosol to the membrane.	[[205], [206], [207], [208], [209]]
		*In vitro* phosphorylation of model substrates/peptides by PKC/PKN (used for crude cell extracts or isolated protein):	
		- MARCKS (myristoylated alanine-rich C kinase substrate) phosphorylation; utilized to evaluate PKC activity in biochemical assays - S6 peptide (AKRRRLSSLRA) - PKC δ peptide (AMFPTMNRRGSIKQAKI or RFARKGSLRQKNVHEVK) - fluorescent peptides	
	direct measurement of PKC/PKN activity *in vivo*	genetically encoded FRET-based reporters that evaluate (spatio)temporal PKC activity:	[206,[210], [211], [212], [213], [214], [215]]
		- CKAR (C kinase activity reporter); tests for activity of PKC isozymes (note that PKN1 phosphorylates the original CKAR *in vitro* as well) - δCKAR; specifically tests for activity of PKCδ - KCP-1 (PKC probe); tests for activity of PKC isozymes - Eevee backbone-based PKC reporter - Newer organelle-targeted variants of the CKAR reporter that specifically evaluate PKC activity at the plasma membrane, Golgi-apparatus, in the cytosol, mitochondria, or nucleus.	
		While no FRET-based reporter constructs that specifically measure PKN activity have been reported, a reporter that utilizes the Rho-binding domain in PKN1 is used to dynamically evaluate cellular Rho-activity.	
	PKC/PKN phosphorylation state	phospho-specific antibodies	[132,136]
		Note that phosphorylation of PKC and PKN in the activation-loop, turn motif or hydrophobic motif may not necessarily be used to evaluate kinase activity per se (e.g. PKC in the autoinhibited confirmation was shown to protect its phosphorylation sites from phosphatase activity).	
Agonists and drugs used in research and clinical trials	pseudosubstrate & peptide inhibitors	Small peptides based on PKC/PKN pseudosubstrates and other kinase segments, as well as kinase scaffolds	[144,152,153,159,160,216]
		- PKCθ pseudosubstrate inhibitor PI used in diabetic cardiomyopathy (Myr-LHQRRGAIKQAKVHHVKC) - caveolin-1 and − 3 scaffolding domain derived peptides inhibit PKCα and PKCζ autophosphorylation - TAT(47–57)-epsilonV1–2 peptide from PKCepsilon tested in I/R and transplantation rejection - cell-permeable PKCζ peptide inhibitor tested in I/R (Myr-SIYRRGARRWRKL) - PKCδ inhibitor KAI-9803 (see below) - PRL peptide inhibitor targeting PKN1 (PRLRRQKKIFSKQQG)	
	Bisindolylmaleimide class of inhibitors	class of PKC inhibitors tested in various cardiomyopathy and heart-failure models	[2,141,142,217,218]
		- bisindolylmaleimide-I (GF109203X, Gö 6850) - Ro-31-8220 - Ro-32-0432	
		Some of these inhibitors have been shown to affect other cellular kinases (e.g. p70S6K, GSK3β)	
	ruboxistaurin (LY333531; Arxxant)	PKCα/β inhibitor (IC_50_ = 4.7–5.9 nM)	NCT02769611 [[137], [138], [139], [140]]
		less selectivity for other conventional and novel PKC's;	
		- tested in various MI models - efficacy to treat heart failure currently tested in phase I/II escalation trials	
	Sotrastaurin (AEB071)	Pan-PKC inhibitor, showing some selectivity for PKCθ	[[145], [146], [147]]
		- tested as immunosuppressant after organ transplantation - used in various clinical trials, but led to increased instances of tachycardia and other adverse events	
	KAI-9803	Selective PKCδ inhibitor peptide derived from the δV1–1 portion of δPKC.	NCT00785954 NCT00093197 [[154], [155], [156]]
		Efficacy and safety to treat myocardial infarction was tested in clinical trials and did not reduce biomarkers of myocardial injury	
	2,4-diaminopyrimidine and 2,4-diamino-5-nitropyrimidine derivatives	PKCθ inhibitors of this class were tested for cardiac allograft rejection in rats and inflammation-induced cardiac and skeletal muscle damage in a dystrophic mouse model	[149,151]
	Rottlerin (Mallotoxin)	originally reported as PKC inhibitor low selectivity, many off target effects	[133]
	Echinochrome A	PKCɩ inhibitor, also exhibiting some selectivity for PKCδ.	[157,158]
		Found to promote cardiomyocyte differentiation from embryonic stem cells.	
	Y27632	PKN2 inhibitor (IC_50_ = 600 nM); also inhibits p160ROCK and PKC	[161]

The importance of the cellular context also plays significant roles when using agonists to positively modulate kinase activity. Phorbol 12-myristate 13-acetate (PMA), a widely used agonist for conventional PKCs results in their depletion after long-term exposure (reviewed and commented in [136]), highlighting the importance to carefully design cell-biological and physiological experiments to ensure activation of the kinase, rather than its degradation.

## 3 Therapeutic approaches

Given the importance of PKCs and PKNs, numerous studies have focused on either developing isoform specific peptide inhibitors, or utilizing gene-modified mice or *in vivo* animal surgery to test the therapeutic value of established small molecule inhibitors.

### 3.1 Conventional PKCs as therapeutic targets

The earliest developed inhibitors targeted mostly conventional PKCs, including Ruboxistaurin (LY333531, Arxxant), which specifically targets PKCα/βΙΙ. Ruboxistaurin was found to partially reverse the cardiac remodeling by improving contractility in a pig model for myocardial infarction [137]. In experimental rodent models, ruboxistaurin administration was similarly beneficial, protecting against cardiac microvascular ischemia reperfusion injury and reversing cardiac microvascular barrier dysfunction [[138], [139], [140]]. Although it has been shown that ruboxistaurin inhibits both, PKCα and PKCβΙΙ, an elegant study clearly demonstrated that the increase in cardiac contractility mediated by ruboxistaurin was mediated through PKCα, not PKCβ [65].

Besides ruboxistaurin, there are a several other conventional PKC inhibitors being tested in various animal models for cardiovascular disease. The importance for PKC activity to the cardiac phenotype observed in a mouse model for myotonic dystrophy type 1 was explored by treating the animals with Ro-31-8220, a PKC inhibitor of the bisindolylmaleimide class. Inhibition of PKC via Ro-31-8220 administration improved the mortality rates, and was associated with better cardiac conduction and contractile function [141]. Both Ro-31-8220 and Ro-32-0432, another bisindolylmaleimide class inhibitor that is orally administrable, are used *in vivo* and in isolated, Langendorff perfused hearts. Both inhibitors can significantly augment cardiac contractility and even restore pump function in MLP knockout mice [2]. In addition to the regular cardiac injury models, another interesting study tested the efficacy of PKC inhibitor Ro-32-0432 in Lewis rats, an experimental autoimmune myocarditis model. Intraperitoneal administration of Ro-32-0432 attenuated the development of the heart failure by repressing hypertrophic marker gene expression as well as alleviating the apoptosis seen in these animals [142].

Lastly, the conventional PKC inhibitor Bisindolylmaleimide-I (GF109203X, Gö6850) has been used to treat the impairment of neurovascular coupling in type I diabetes mellitus in rats [143] as well as dilated cardiomyopathy and heart-failure in MLP knockout mice [42].

### 3.2 Inhibiting novel PKCs in heart disease and after heart transplantation

Although novel PKC family members are not as abundantly expressed in the heart as conventional PKCs, their inhibitors have also been tested to treat cardiovascular disease, as well as for heart transplantations.

One example is a modified PKCθ peptide inhibitor PI, which was used to treat diabetic cardiomyopathy in a model of streptozotocin induced type-1 diabetes. Inhibition of PKCθ using PI significantly attenuated the cardiac fibrosis response in this animal model and lead to slightly improved contractile force generation [144].

Another example of a pan-PKC inhibitor that shows some selectivity for PKCθ is sotrastaurin (AEB071). Several animal studies used this inhibitor as immunosuppressive drug during heart transplantation, resulting in lower transplant rejection and increased survival rates [145,146]. Sotrastaurin was used in several clinical trials, mostly as immunosuppressive after organ transplantation or to treat various cancer types. However, published results on one of these trials indicate increased incidents of tachycardia and other adverse events in the treatment group, leading to a premature stop of the study [147]. Further studies are required to fully evaluate the efficacy and safety of this inhibitor, as flaws in this prematurely aborted study may have unintentionally skewed results [148].

Other studies investigating cardiac transplant rejection and cardiac muscle structure in dystrophic mice identified and characterized novel derivatives of 2,4-diaminopyrimidine and 2,4-diamino-5-nitropyrimidine as selective inhibitors against PKCθ [[149], [150], [151]]. Treatment with these compounds reduced inflammation in dystrophic mice, and prolonged graft survival in an *in vivo* rat heterotopic cardiac transplant model. A similar study was performed using peptide inhibitors of PKCε. To determine whether sustained inhibition of PKCε can improve the success of cardiac transplantation, a PKCε inhibitor peptide (TAT(47–57)-epsilon V1–2) was delivered in an FVB mouse heterotopic transplantation model [152,153]. Results from these experiments suggested PKCε activity contributes at least partially to the chronic immune response after cardiac allograft, and inhibition of PKCε could provide a novel therapeutic strategy for prolonged graft survival.

### 3.3 Atypical PKCs as therapeutic target

Perhaps even less is known for the inhibitors that can specifically target atypical PKC family proteins. Among them, rottlerin, the most widely reported PKCδ inhibitor, has been used in a variety of studies. However, aforementioned problems with the specificity of this inhibitor need to be taken into account when evaluating experimental results [133]. KAI-9803, a more selective PKCδ inhibitor peptide derived from the δV1–1 portion of PKCδ was tested in clinical trials to treat myocardial infarction and was found not to reduce biomarkers of myocardial injury [[154], [155], [156]].

Echinochrome A, a naphthoquinoid pigment from sea urchins [157] that inhibits PKCɩ promoted mouse embryonic stem cells differentiation into cardiomyocytes [158]. *In vitro* kinase assays using echionochrome A also reported decreased activity of PKCδ (IC_50_ = 107 μM). However, the detailed inhibitory mechanism of echionochrome A, as well as its selectivity towards other PKCs has yet to be explored.

Lastly, a 13-amino acid PKCζ inhibitor peptide was used to treat I/R injured hearts [159]. Inhibition of PKCζ significantly attenuated polymorphonuclear leukocytes induced cardiac dysfunction during I/R, suggesting the functional importance of PKCζ in cardiac injury.

### 3.4 PKN inhibitor therapeutics

Unlike the numerous inhibitors targeting for PKC family members, the effort for developing selective inhibitors for protein kinase N remains sparse. One such example is an intracellularly acting inhibitory peptide based on the C-terminal part of the C2-like domain in PKN1 [160]. This small 15 amino acid long peptide (PRL) and its modified derivatives can selectively inhibit the kinase activity of all PKN isoforms with a K_i_ = 0.7 μM. Another screening effort identified a number of PKN inhibitors with various isoform selectivity [161]. Specifically, this study demonstrated that Y27632 can inhibit all PKN isozymes. Interestingly, this report also suggested that some of the more widely used PKC inhibitors, including Ro 31–8220 and bisindolylmaleimide-I, are also able to target PKN1 and PKN2.

### 3.5 Lessons from clinical trials

Although PKCs and PKNs are promising targets whose therapeutic potential has been demonstrated in numerous animal models, many of the clinical trials that are currently carried out to determine their potential, efficacy and safety remain undetermined. Ruboxistaurin, one of the best-known PKC inhibitors was used to test the therapeutic potential for the treatment of diabetic peripheral neuropathy and retinopathy. Ruboxistaurin (Arxxant) was introduced by Eli Lilly and Company, and the FDA advised the company in 2006 that it will require efficacy and safety data from an additional Phase 3 study before it will consider approving the molecule. These additional requirements were partially based on insufficient evidence for the drug's efficacy, and results from a clinical trial (study B7a-LC-MBDT) showing that the inhibitor led to a small but significant prolongation of the QT-interval in treated patients, specifically in females [162]. Further *in vitro* experiments indicated that ruboxistaurin is able to block the hERG channel (human ether-a-go-go related gene) with an IC_50_ of 35.6 nM, altering the repolarization of the myocardium and potentially contributing to the observed QTc prolongation [162]. However, additional studies using canine Purkinje fiber preparations, and *in vivo* studies in large animals (dogs) resulted in contradictory data. Moreover, there was concern for an increase in creatine phosphokinase levels in a large animal study, as well as possible interactions with statins and adverse effects on muscle function. Ultimately, the withdrawal assessment report of the European Medicines Agency concludes that “There were a number of concerns related to safety. The QTc related issues [of ruboxistaurin] were considered [a] major safety concern. The development of diabetic retinopathy is a slow process and takes many years while adverse implications of drug prolonging QT is immediate (VT and sudden death).” [162] Consequently, the drug approval application was subsequently withdrawn in Europe and the US. However, additional trials are currently underway to test efficacy of ruboxistaurin in heart failure (NCT02769611) and other conditions.

## 4 Conclusion

Kinases of the PKC and PKN families play crucial roles in the heart. More basic and clinical research is needed to fully understand their complex cardiac specific signaling, influence of posttranslational modifications, crosstalk within the PKC and PKN families as well as other signaling pathways, their cardiac specific substrates and the influence they exert on cardiac physiology and modulation of the gene program during remodeling.

While their role in cardiovascular disease represents an alluring therapeutic target for many disorders, multiple issues with the usage of PKC/PKN inhibitors remain unresolved. These include off-target effects on other kinases and proteins (e.g. ion channels), and problems stemming from systemic inhibitor action on PKC/PKN in other organs and tissues, where their inhibition may lead to unwanted adverse side effects. These need to be specifically taken into account where long-term treatment of patients is required, e.g. in heart failure. In addition, lessons from pre-clinical trials performed in rodent models for cardiac diseases, and results from gene-targeted mice for members of the PKC/PKN kinase families may only be partially translatable to human pathophysiology. Examples include distinct species-specific differences in human ERG channel physiology compared to their rodent analogs [163], and the fact that specific roles for kinase functions have been largely defined by loss/gain of function studies using murine models.

## 5 Methods

### 5.1 Biostatistics

Evolutionary analyses of PKC and PKN kinase domains was conducted using MEGA7 [164,165]. The evolutionary history was inferred by using the Maximum Likelihood method based on the JTT matrix-based model. The tree with the highest log likelihood is shown. Initial tree(s) for the heuristic search were obtained automatically by applying Neighbor-Join and BioNJ algorithms to a matrix of pairwise distances estimated using a JTT model, and then selecting the topology with superior log likelihood value.

### 5.2 Human cardiac biopsies

Human Research Ethics Committee approval was obtained by St Vincent's Hospital (#H03/118; 2017 renewal #7326), and by the University of Sydney Human Research Ethics Committee (#12146, #159401). Human tissue was used in accordance with the ethical guidelines of King's College London (College Research Ethical Committee 04/05–74; REC reference 12/EM/0106), under current UK and EU laws. Collection and analysis of cardiac biopsies from left ventricular free walls of patients was done as described before [42].

### 5.3 Animals

Animals used in this study were described in detail [42]. For protein analysis, samples from the left ventricular free wall of hearts from 6–9-month-old adult male and female mice were used. All procedures were reviewed and approved by the Animal Care and Use Committee at the University of California San Diego.

### 5.4 Protein expression analysis and antibodies

Total protein extracts of hearts for immunoblot analysis were generated by homogenizing samples directly into SDS-sample buffer (BioRad) using a polytron blade homogenizer (Pro Scientific Inc). Normalized total protein extracts were run on uniform 15% acrylamide SDS-PAGE gels (BioRad, Invitrogen), followed by immunoblotting on nitrocellulose membranes (BioRad). Following transfer, the membranes were stained with Ponceau Red, blocked with 5% Bovine Serum Albumin (Sigma) in Low Salt Buffer (0.9% NaCl, 9 mM Tris pH 7.4, 0.1% Tween-20) and incubated with the appropriate primary and secondary antibodies with intermittent washing in Low Salt Buffer. Results from the chemiluminescence reaction were visualized using X-ray films. Primary antibodies used in this study were PLN-Ser10 (Badrilla, A010-10AP), total phospholamban antibody (Badrilla, A010–14), SERCA2 (Cell Signaling, 9580), cardiac actin (Progen Biotechnik, clone Ac1–20.4.2), GAPDH (Santa Cruz Biotechnology, clone 6C5).

### 5.5 GST-construct generation and *in vitro* kinase assays

Generation and purification of GST-phospholamban constructs was done as described previously [42]. For *in vitro* kinase assays, 2 mg of wildtype or S10A mutant GST-PLN(N) (cytoplasmic domain of mouse phospholamban; amino acids 1-34; NM_023129) were combined with purified PKCα (10 ng/reaction, P0329, Sigma-Aldrich) in kinase buffer (20 mM HEPES KOH pH 7.4, 10 mM MgSO_4_, 0.1 mM CaCl_2_). The mix was supplemented with unlabelled ATP (20 mM) and a combination of 1 μg/μl phosphatidylserine and 2 μg/μl diacylglycerol, dissolved in resuspension buffer (10 mM HEPES pH 7.4, 0.3% Triton X-100). Following incubation at 30 °C for 30 min, samples were mixed with SDS-sample buffer (BioRad) and separated using SDS-PAGE, followed by immunoblot analysis, as described above.

## Conflict of interest

None.
